# High CRP-albumin ratio predicts poor prognosis in transplant ineligible elderly patients with newly diagnosed acute myeloid leukemia

**DOI:** 10.1038/s41598-022-12813-1

**Published:** 2022-05-25

**Authors:** Hajime Senjo, Masahiro Onozawa, Daisuke Hidaka, Shota Yokoyama, Satoshi Yamamoto, Yutaka Tsutsumi, Yoshihito Haseyama, Takahiro Nagashima, Akio Mori, Shuichi Ota, Hajime Sakai, Toshimichi Ishihara, Takuto Miyagishima, Yasutaka Kakinoki, Mitsutoshi Kurosawa, Hajime Kobayashi, Hiroshi Iwasaki, Daigo Hashimoto, Takeshi Kondo, Takanori Teshima

**Affiliations:** 1 Department of Hematology, Faculty of Medicine, Hokkaido University, N15 W7, Kita-ku, Sapporo, 060-8638 Japan; 2 Department of Hematology, Sapporo Hokuyu Hospital, Sapporo, Japan; 3 Department of Hematology, Sapporo City General Hospital, Sapporo, Japan; 4 Department of Hematology, Hakodate Municipal Hospital, Hakodate, Japan; 5 Department of Hematology, Tonan Hospital, Sapporo, Japan; 6 Department of Hematology, Japanese Red Cross Kitami Hospital, Kitami, Japan; 7 Blood Disorders Center, Aiiku Hospital, Sapporo, Japan; 8 Department of Hematology, Teine Keijinkai Hospital, Sapporo, Japan; 9 Department of Hematology, Kin-Ikyo Chuo Hospital, Sapporo, Japan; 10 Department of Hematology, Kushiro Rosai Hospital, Kushiro, Japan; 11 Department of Hematology, Asahikawa City Hospital, Asahikawa, Japan; 12 Department of Hematology, Hokkaido Cancer Center, Sapporo, Japan; 13 Department of Hematology, Obihiro Kosei General Hospital, Obihiro, Japan; 14 Department of Hematology, Sapporo Kosei General Hospital, Sapporo, Japan

**Keywords:** Acute myeloid leukaemia, Prognostic markers

## Abstract

Acute myeloid leukemia (AML) patients older than 65 years have a poor prognosis. Recently, CAR (C-reactive-protein/albumin ratio) has been actively reported as a prognostic index reflecting the nutritional and inflammatory status of elderly patients with solid tumors, but the usefulness of this index as a prognostic indicator in transplant-ineligible elderly AML patients has not been investigated. We studied genetic alterations and CARs in 188 newly diagnosed AML patients aged 65 years or older who were treated in a multicenter setting and had treated without HSCT. Both NCCN 2017 risk group, reflecting the genetic component of the tumor, and CAR, reflecting the inflammatory and nutritional status of the patient, successfully stratified the overall survival (OS) of the patients (2-year OS; CAR low vs high, 42.3% vs 17.8%, *P* < 0.001). Furthermore, in multivariate analysis, NCCN 2017 poor group and high CAR were extracted as independent poor prognostic factors predicting 2-year OS in the current study. We found, for the first time, that CAR at diagnosis predicted the prognosis of elderly patients with newly diagnosed AML treated without HSCT.

## Introduction

Patients aged 65 years or older who are first diagnosed with acute myeloid leukemia (AML) generally have a poor prognosis^1^. One of the reasons for this is that many of these patients are ineligible for hematopoietic stem cell transplantation (HSCT) due to barriers such as age-related loss of organ reserve. Although a large proportion of patients with first-episode AML are 65 years of age or older, the prognostic indicators for newly diagnosed AML currently used in clinical practice are mostly established from data on younger patients, and their feasibility for elderly patients needs to be carefully considered in different cases. Large-scale analysis of real-world data is needed to establish appropriate prognostic indicators for cases in elderly patients with newly diagnosed AML. The prognostic risk classification based on the National Comprehensive Cancer Network (NCCN) Guidelines Version 3 (2017; NCCN 2017), which reflects genetic alteration of tumors^2^ is highly useful in clinical practice and commonly used (Table S1). We had previously reported that this classification is as useful in older patients with newly AML as it is in younger adults^3^. We also reported in the same report that a new nutritional index, the simple CONUT score (sCONUT score), which can be calculated based solely on serum albumin and total cholesterol (T-chol) levels, excluding the total lymphocyte count from the conventional CONUT score, is useful in predicting the prognosis of elderly patients with newly diagnosed AML at diagnosis^3^. At the same time, we mentioned about limitations of the study such as lack of biochemical data and/or comorbidities including inflammatory status at diagnosis in some patients. Besides, it was demonstrated that the prognosis of patients with poor, intermediate, and favorable risk group by NCCN 2017 classification could not be further divided with statistically significance by sCONUT score. One possible reason for the difficulty in predicting prognosis based on nutritional indices alone was the lack of assessment of inflammation. This is because many elderly patients who are deemed ineligible for HSCT had comorbidities and/or reduced organ reserve. Inflammatory complications, such as infections, are the most frequent cause of ineligibility for transplantation. Hence, we suspected that more feasible prognostic score which reflects not only the nutritional factor but also the inflammatory status is needed for evaluation of elderly patients with newly diagnosed AML. While the inflammatory and nutritional status assessment using C-reactive-protein (CRP) to albumin ratio (CAR) based on serum levels of CRP and albumin (Alb) has been shown to predict prognosis of elderly patients with solid tumors^4–7^ and hematological malignancies treated with HSCT^8^, its prognostic significance in elderly patients with AML remains to be clarified. In the current study, we aimed to determine the prognostic value of CAR in elderly patients with newly diagnosed AML.

## Patients and methods

### Patients

The Hokkaido Leukemia Network (HLN) prospectively collects AML samples from hospitals of the North Japan Hematology Study Group (NJHSG)^3, 9^. This study focused on new AML patients aged 65 years or older who had not received a HSCT and investigated cytogenetic and leukemic cell molecular abnormalities (FLT3-ITD, NPM1, CEBPA, KIT, etc.)^3, 9^. Based on the NCCN 2017, patients were stratified into favorable, intermediate, and unfavorable risk groups^2^. A total of 517 AML patients were enrolled in the HLN from 2010 to 2019, and 188 patients aged 65 years or older who were treated without HSCT were included (Fig. S1). One hundred and eighty-eight patients aged 65 years or older who received the treatment were enrolled in the study (Fig. S2). Most of the cases included in this study are in the same population as those in studies we have reported in the past focusing on elderly nutritional scoring^3^.

This study was conducted in compliance with ethical principles based on the Declaration of Helsinki and was approved by the Institutional Review Board of Hokkaido University Hospital (No. 015-0344). Written consent was obtained from each patient for participation in the study.

### Study objections

In addition to gender and age, we collected information on each patient at diagnosis of AML; blood sample tests (CRP (mg/dL), Alb (g/dL), T-Chol (mg/dL), estimated glomerular filtration rate; eGFR), the presence of fever over 38 °C, the presence of complications at diagnosis (active infection, other active malignancy, collagen disease). For post-diagnostic information, we collected initial treatment, and the effect of treatment on initial treatment were observed for each patient. The relationship between these objectives and prognosis was then examined.

### Risk indices

CAR was calculated from CRP (mg/dL) and Alb (g/dL) as previously reported^8^. Based on the result of blood sample test, we assessed CAR [CRP (mg/dL)/Alb (g/dL)] at diagnosis for each patient. We performed Receiver Operatorating Characteristic (ROC) analysis of CAR, CRP, and Alb^−1^ with death as a positive event. We obtained cutoff values of CAR 1.1, CRP 2.0 (mg/dL), and Alb 3.3 (g/dL) (Fig. S3A–C). Using these cutoff values, patients were classified as high and low, respectively. We evaluated the impacts of NCCN 2017 and CAR on overall survival (OS) in these patients.

### Statistical analysis

The method of statistical study is the same as in our previous reports^3, 9^. OS was calculated as the time from the date of diagnosis to death or last follow-up; the probability of OS was estimated using the Kaplan–Meier method, and differences between patient groups were analyzed using the log-rank test. Patient characteristics at baseline were tabulated to check for imbalances in demographic information. The variables evaluated in the univariate analysis were all items listed in Table 1. Risk factors for OS at diagnosis were assessed by multivariate Cox regression. The Mann–Whitney *U* test was used to compare data between groups. Differences in treatment were assessed by the X^2^ test; all P values were two-sided, with a P value of 0.05 as the cutoff value for statistical significance. As already mentioned, the above statistical analysis used the same methodology as in our previous reports^3, 9^. All statistical analyses were performed using EZR software^10^ (version 1.50, http://www.jichi.ac.jp/saitama-sct/SaitamaHP.files/statmedEN.html).

**Table 1:** Patient characteristics and univariate analysis of the risk factors associated with 2-year OS.

Characteristics	No. (%)	OS (%)	*P* value
**Sex (n = 188)**			0.144
Male	119 (63.3)	27.7	
Female	69 (36.7)	39.1	
**Age (n = 188)**			0.037
65–70	74 (39.4)	42.7	
71–80	83 (44.1)	44.1	
81–	31 (16.5)	16.5	
Median (range)	72 (65–93)		
**NCCN 2017 (n = 188)**			< 0.01
Favorable	45 (23.9)	58.1	
Intermediate	101 (53.7)	29.4	
Poor	42 (22.3)	12.6	
**Alb level (n = 188)**			0.054
≥ 3.3 g/dL	114 (60.6)	36.6	
< 3.3 g/dL	74 (39.4)	26.6	
Median (range), g/dL	3.5 (1.6—5.9)		
**T-Chol level (n = 144)**			0.085
≥ 140 mg/dL	61 (42.4)	36.7	
< 140 mg/dL	83 (57.6)	23.4	
Median (range), mg/dL	140 (74–248)		
**CRP (n = 188)**			0.159
≥ 2.0	94 (50.0)	26.5	
< 2.0	94 (50.0)	37.2	
Median (range), mg/dL	2.0 (0.01–35.39)		
**Simplified CONUT score (n = 144)**			0.011
Low	82 (56.9)	38.9	
High	62 (43.1)	13.8	
**CAR (n = 188)**			< 0.01
Low	113 (60.1)	47.8	
High	75 (39.9)	20.0	
**Fever ≥ 38.0 °C (n = 188)**			0.385
Yes	41 (21.8)	17.1	
No	147 (78.2)	21.8	
**eGFR (n = 188)**			0.605
≥ 90	13 (6.9)	38.5	
65–89	78 (41.5)	51.3	
45–59	46 (24.5)	35.5	
30–44	38 (20.2)	26.4	
15–29	9 (4.8)	18.8	
< 15	4 (2.1)	0.0	
Median (range), ml/min/1.73 m^2^	74.8 (9.27–124.5)		
**Active infection (n = 188)**			0.840
Yes	50 (26.6)	22.0	
Pneumonia	46 (24.5)		
Oral mucositis	4 (2.1)		
Enteritis	2 (1.1)		
Bacteremia	1 (0.5)		
Liver abscess	0		
Skin and soft tissue infection	0		
No	138 (73.4)	20.3	
**Other active malignancy (n = 188)**			0.671
Yes	8 (4.3)	25.0	
Prostate cancer	2 (1.1)		
Pancreatic cancer	2 (1.1)		
Small cell lung cancer	1 (0.5)		
Colon cancer	1 (0.5)		
Oropharyngeal cancer	1 (0.5)		
Multiple myeloma	1 (0.5)		
No	180 (95.7)	20.6	
**Collagen disease (n = 188)**			1.0
Yes	5 (2.7)	20.0	
Rheumatoid arthritis	3 (1.6)		
Polymyalgia rheumatica	1 (0.5)		
Sjögren's syndrome	1 (0.5)		
No	183 (97.3)	20.8	
**Treatment (n = 188)**			0.583
IDR + AraC	52 (27.7)	37.6	
DNR + AraC	27 (14.4)	48.3	
DNR + BHAC	21 (11.2)	5.6	
CAG	25 (13.3)	36.0	
AZA	18 (9.6)	33.3	
Low-dose AraC	7 (3.7)	37.2	
Other chemotherapy	15 (8.0)	25.0	
Best supportive care	23 (12.1)	0.0	
**Response (n = 181)**			< 0.01
CR	62 (33.0)	38.9	
Non-CR	119 (63.3)	15.1	

*CRP* C-reactive protein, *TLC* total lymphocyte count, *Alb* albumin, *T-Chol* total cholestero, *IDR* idarubicin, *AraC* cytarabine, *DNR* daunorubicin, *BHAC* enocitabine, *CAG* low-dose cytarabine, aclarubicin hydrochloride, and granulocyte colony stimulating factor; *AZA* azacitidine, *CR* complete remission, *ND* no data.

The authors confirm that this study was conducted in accordance with relevant national, international, and institutional guidelines. The datasets used and/or analyzed during the current study available from the corresponding author on reasonable request.

## Results

### Patient characteristics

Baseline patient characteristics were listed in Table 1. A median patient age at diagnosis was 72 years, ranging from 65 to 93 years. We identified cytogenetic and molecular abnormalities in leukemic cells, including FLT3-ITD, NPM1, CEBPA, and KIT at diagnosis in all patients. Based on the results, we classified the risk of patients according to the NCCN 2017 stratification^2^ (Table S1). Then, in the current study, 24%, 54%, and 22% of patients were classified as having favorable, intermediate, and poor risk, respectively.

**Figure 1:**
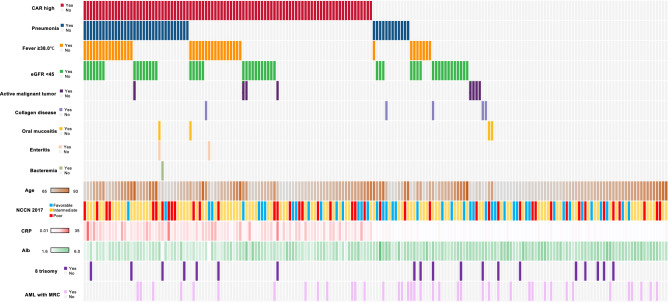
Distribution of the backgrounds and complications presented by the patient.

**Figure 2:**
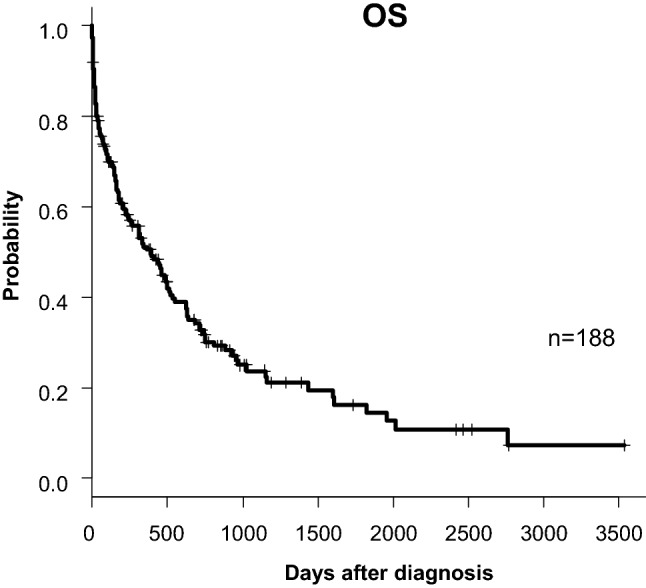
Kaplan–Meier plots of overall survival (OS) of patients for the entire cohort.

Blood sample tests at diagnosis for all 188 elderly patients not eligible for transplantation were available for analysis. The median serum Alb level was 3.5 g/dL, with a range of 1.6 to 5.9 g/dL. Body temperature at diagnosis was available in 188 patients and 21.8% of the patients had fever with 38.0℃ or higher. The median serum CRP level at diagnosis was 2.0 mg/dL, with a range of 0.01 to 35.39 mg/dL. The median serum T-chol level was 140 mg/dL, with a range of 74–248 mg/dL in only 144 patients, because some patients were not evaluated serum T-chol levels at diagnosis. Serum creatinine (Cre) levels at diagnosis were available in 188 patients with 0.85 mg/dL as median level, ranging from 0.40 to 4.51 mg/dL. eGFR at diagnosis was calculated with 74.8 ml/min/1.73 m^2^ as median, ranging from 9.27 to 124.5 ml/min/1.73 m^2^. To identify the causes of CRP elevation, we retrospectively investigated if patients had typical inflammatory complications; active infections, other active malignancies, and collagen disease. For active infections, 46 patients were diagnosed to have pneumonia, 4 patients had oral mucositis, 2 patients had enteritis, and 1 patient had bacteremia. No patients showed findings of liver abscess and/or skin & soft tissue infection at diagnosis in this cohort. As above, fifty patients (26.6%) had complications of active infections. For other active malignancy, 8 patients had diagnosed concurrent malignancies: prostate cancer in 2 patients, pancreatic cancer in 2 patients, small cell lung cancer, colon cancer, oropharyngeal cancer, and multiple myeloma. The remaining 180 patients had no other active malignancy as complication. For collagen disease, 3 patients had active rheumatoid arthritis, one with polymyalgia rheumatica, and one with Sjögren’s syndrome. Induction therapy based on decision of each physician varied according to risk factors of the disease and general status of patients. As shown in Table 1, 87.9% of the patients were initially treated with chemotherapy. The remaining 12.1% of the patients were treated with best supportive care. Distribution of the backgrounds and complications presented by the patient is shown in Fig. 1. Overall, 62 patients achieved complete remission (CR) (33%; Table 1). The median OS was 238 days, and the 5-year OS was 16.2% (Fig. 2).

### The prognostic risk classification based on NCCN 2017

**Figure 3:**
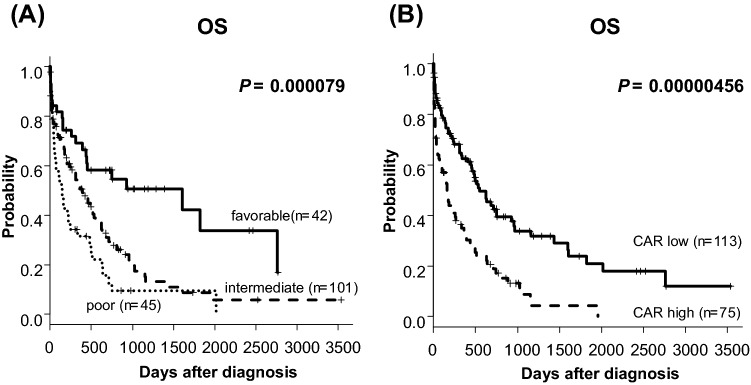
Kaplan–Meier plots of OS according to NCCN 2017 risk classification (**A**) and CAR (**B**).

The risk classification based on NCCN 2017 successfully stratified the OS of the patients (5-year OS; favorable group, 33.7% vs intermediate group, 8.74% vs poor group, 9.44%, *P* = 0.000079, Fig. 3A).

### CAR as a prognostic biomarker

We first analyzed the impacts of serum levels of CRP and albumin at diagnosis as prognostic indicator. In this cohort, neither serum levels of CRP nor albumin did not stratify the prognosis significantly (5-year OS; CRP; 18.7% vs 16.2%, *P* = 0.0618, Alb; 19.0% vs 13.4%, *P* = 0.0876, Fig. S3E,F). For CAR, OS was significantly lower in patients with high CAR than in those with low CAR (5-year OS; 23.8% vs 4.37%, *P* = 0.00000456, Fig. 3B).

### Overlapping factors for CAR elevation

**Figure 4:**
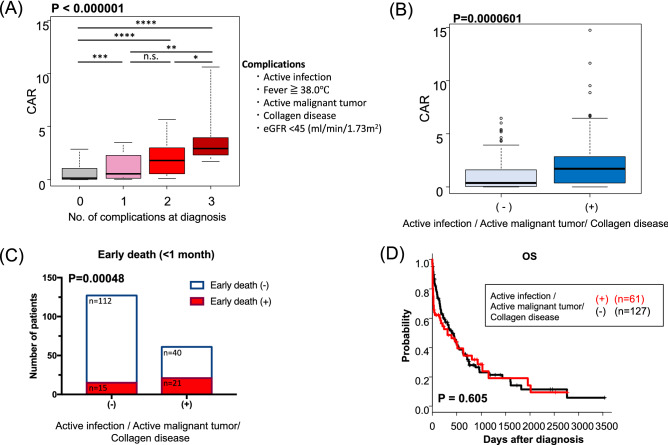
(**A**) Distribution of CAR in patients without complications (n = 63) and 1 complication (n = 76), 2 complications (n = 34), 3 complications (n = 15) at diagnosis. (**B**) Distribution of CAR in patients with (n = 61) or without (n = 127) active infection and/or active malignant tumor and/or collagen disease at diagnosis. (**C**) The number of patients died within 1 month from diagnosis in patient groups with/without active infection and/or active malignant tumor and/or collagen disease at diagnosis. (**D**) Kaplan–Meier plots of OS according to patient groups with/without active infection and/or active malignant tumor and/or collagen disease at diagnosis (*P < 0.05; **P < 0.01; ***P < 0.005; ****P < 0.001, n.s., not significant).

To identify the causes of the elevated CAR (i.e. high CRP and/or low Alb), we focused on active infections, fever of 38.0 °C or more, active malignant tumor, collagen disease, and low eGFR < 45 ml/min/1.73 m^2^ at diagnosis as typical complications effecting high CAR. Since many patients had more than one complication at diagnosis (Fig. 1), we investigated the association between CAR and the number of complications at diagnosis. We found that the patients with the larger number of complications at diagnosis showed high CAR (P < 0.000001, Fig. 4A). Next, we specifically focused on active infection, active malignant tumor, and collagen disease as complications causing inflammatory conditions. Patients with any one of these three complications had higher CAR (P = 0.0000601, Fig. 4B). Additional analysis revealed that patients with one or more of these three complications had higher risk for early deaths within 1 month from diagnosis (P = 0.00048, Fig. 4C), but their long-term prognosis was not stratified with those without complications (P = 0.605, Fig. 4D).

### Usefulness of CAR in patients without inflammatory complications

**Figure 5:**
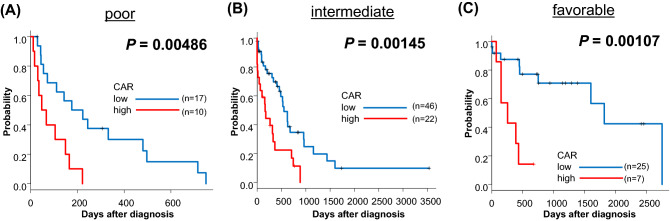
Kaplan–Meier plots of OS according to CAR in patients without 3 complications (active infection/active malignant tumor/collagen disease) at diagnosis, and categorized with poor risk (**A**), intermediate risk (**B**), and favorable risk (**C**) stratified by NCCN 2017 risk classification.

As the patients with active infection, active malignant tumor, and collagen disease had high CAR at diagnosis (Fig. 4B), we then excluded patients with these comorbidities and investigated if CAR could further stratify risks of the patients in each NCCN 2017 risk group. We found that patients with high CAR had significantly poorer prognosis than patients with low CAR in each risk group of NCCN 2017 (CAR low vs high, 2-year OS; poor; 7.50% vs 0.0%, *P* = 0.00486, 5-year OS; intermediate; 9.91% vs 0.0%, *P* = 0.00145, favorable; 42.4% vs 0.0%, *P* = 0.00107, respectively, Fig. 5A–C).

### Univariate and multivariable analyses of clinical prognostic factors

We analyzed various prognostic factors for OS (Table 1). In a univariate analysis, Alb level, T-chol level, eGFR and CRP were not associated with poor 2-year OS. On the other hand, higher age, poor risk group based on NCCN 2017 classification, high sCONUT score, high CAR, and not reached CR after first induction chemotherapy were associated with poor 2-year OS. We therefore performed multivariable analysis that included higher age, poor risk group based on NCCN 2017 classification, high CAR, and non-CR. In this multivariable analysis, poor group in NCCN 2017 risk classification, high CAR and non-CR were independently associated with poor 2-year OS (Age; HR, 1.77; 95% CI 1.11 to 2.82, *P* = 0.016, NCCN 2017; HR, 1.65; 95% CI 1.14 to 2.40, *P* = 0.008668, non-CR; 3.88; 95% CI 2.36 to 6.38, *P* = 0.00000008393, CAR; HR, 1.58; 95% CI 1.09 to 2.28, *P* = 0.016; log-rank, Table 2).

**Table 2:** Multivariable analysis of the risk factors associated with 2-year OS.

Characteristics	Hazard ratio	95% CI	*P* value
Age more than 80	1.77	1.11–2.82	0.016
Poor group in NCCN 2017	1.65	1.14–2.40	< 0.01
Non-CR	3.88	2.36–6.38	< 0.01
High CAR	1.58	1.09–2.28	0.016

## Discussion

Annual incidence of AML increases with age and this disease is most common in patients older than 65 years^11^ (Fig. S1). For young adults under 65 years of age with AML, the combination of cytarabine and anthracycline achieves a complete remission rate of 78%, followed by consolidation therapy with a 5-year survival rate of 48%^12, 13^. However, it is often difficult for elderly patients to receive intensive chemotherapy as younger patients^14^. But if left untreated, elderly AML patients usually die within 2 weeks to 2 months from complications such as sepsis and cerebral hemorrhage. Appropriate prognostication is necessary because some elderly patients with elderly AML survive intensive chemotherapy and achieve long survivals, improvements in quality of life and physical function^15^.

We previously reported about a newly developed nutritional status assessments using sCONUT score; the combinational score based on serum level of Alb and T-chol, which successfully stratified the prognosis of the elderly patients with AML^3^. However, after classified into 3 risk groups by NCCN 2017 classification, sCONUT score did not divide the prognosis of the patients with statistically significance in each risk^3^. And as a routine practice, serum level of T-chol was not always evaluated at the initial diagnosis of AML. Additionally, referring to a table would be needed to calculate sCONUT and this score is not seemed to be best suited for quick use in daily clinical practice.

Recently, CAR has been shown to predict prognosis in patients with solid tumors^4–7^ and hematological malignancies treated with HSCT^8^. However, the prognostic value of CAR in elderly AML treated without HSCT remained to be determined. In the current study, we showed that CAR can predict the prognosis of the elderly patients with newly diagnosed AML independent from NCCN disease risk score based on genetic alteration. Moreover, in patients without inflammatory complications at AML diagnosis, it should be noted that patients with each risk group by NCCN 2017 classification were further divided into 2 different prognostic groups with statistical significance (Fig. 5A–C). It is quite meaningful because more precise prognostic prediction based on inflammatory and nutritional status would directly affect the treatment choice of physicians treating patients in this group. It is certain that sCONUT score is a useful prognostic score based on the result of blood sample test at diagnosis. However, compared to sCONUT score, CAR is no doubt much more simple, easier to calculate even with mental arithmetic, conventionally available, and stronger prognostic predictor which can compensates NCCN disease risk efficiently. Moreover, serum levels of albumin and CRP are both always included in initial evaluation of newly diagnosed AML. Despite the nature of retrospective analysis, serum levels of CRP and albumin were available in all 188 cases in the current study. This score would be esteemed for its ease of use in clinical practice.

Recently, Ballo et al. has been reported that the severity of AKI based on KDIGO stratifies the prognosis of patients with newly diagnosed AML who treated with intensive induction therapy^16^. Simple comparison between this report and our study should not be made because the patient background of this report is quite different from our cohort; more than half of the patients were younger than 60 years old, and some of patients had undergone HSCT in their study. However, the result that renal dysfunction would be a strong prognostic indicator is common. In patients with concurrent infections, basal renal dysfunction may interfere with the treatment of infections with various antibacterial and antifungal agents, leading to inadequate treatment and death from infections, which account for most deaths in leukemia patients. Whether directly or indirectly through treatment of infection, renal dysfunction has a significant impact on prognosis of patients with hematological malignancies. In the present study, we retrospectively investigated the presence of concurrent inflammatory complications, active infection, active other malignancy, and collagen disease at AML diagnosis. Importantly, CAR significantly stratified prognosis in this population and the usefulness of CAR as a valid prognostic indicator was retained. These results support the possibility that CAR may be more useful for prognostic stratification of transplant-ineligible elderly patients with AML as an indicator that comprehensively reflects elevated CRP due to causes other than inflammatory complications and decreased Alb due to poor nutritional status or more, not limited to only renal dysfunction.

At the same time, the exact mechanism underlying the usefulness of CAR is unclear and might be complicated. CRP is one of the most useful acute-phase proteins for assessing inflammation. CRP is synthesized in liver in response to IL-6. Several reports showed that high CRP levels are significantly associated with higher non-relapse mortality and poor OS in patients with hematological malignancies treated with HSCT^17–19^. However, the mechanisms underlying the relationship between inflammation and cancer survival have not been elucidated completely. Systematic inflammatory response plays an important role in tumor progression^20^. Inflammatory cells can be tumor promoters with proinflammatory cytokines and the formation of an inflammatory microenvironment^21^. Then, it might be possible that elevated CRP level reflects the high progressive potential of leukemic cells which results in poor prognosis of the patients. It might be possible that leukemic cells itself secrete cytokines that cause elevated CRP, or that high CRP is a surrogate marker for active secretion of cytokines that lead to leukemic cell proliferations.

Historically, there have been reports about the association between hypoalbuminemia and poor prognosis^22–25^. Although the underlying mechanism is not clear, it has been reported that albumin levels decline in older age, and age-related visceral protein loss and/or worsening nutritional status are thought to be the factors behind hypoalbuminemia^25–28^. These factors might also contribute to low albumin levels and poor prognosis in elderly patients with newly diagnosed AML in our cohort.

Notably, neither serum levels of CRP nor albumin alone did not stratify the prognosis significantly (Fig. S3E,F) but combination of these two biomarkers, CAR, successfully divide the prognosis of the patients significantly in the current study. Moreover, our result implies that CAR is an indicator which reflects multiple factors in a single patient, and patients with high CAR at diagnosis might have a poor prognosis due to multiple host factors rather than a single factor. Miyashita et al. mentioned about the possibility that elevated CRP levels and hypoalbuminemia indicate the presence of latent infections and/or occult tissue injuries before conditioning in the patients with hematological malignancies treated with HSCT, and this can also be a possible explanation for elderly AML patients treated without HSCT in our cohort^8^. Interestingly, CAR can significantly divide the prognosis of the patients without inflammatory complications at diagnosis. We often experience AML patients with high CRP despite the absence of infection, other malignant tumor, and collagen disease. The fact that AML patients without these complications and high CAR showed poor prognosis implies that CAR reflects active inflammation status not only caused by infection/other malignant tumor/collagen disease but also caused by the leukemia itself. Furthermore, CAR might reflect underlying inflammation status and nutritional condition of the patient.

Our study has some limitations. Our cohort has limited sample size and lack of data about consolidation therapy due to questionnaire-based retrospective data collection. Although we should also differentiate between AKI and CKD in our analysis, we only had data on blood tests at the time of diagnosis and were unable to calculate serum creatinine levels over time, which is a diagnostic criterion for AKI, and urinary albumin levels, which is a diagnostic criterion for CKD. Our real-world cohort includes heterogeneous patients with various comorbidities. It is obvious that such complications and comorbidities would have a huge impact on the inflammatory and nutritional status. Further investigation for comorbidity is needed in the future studies. At least, it was demonstrated that high CAR at diagnosis is a poor prognostic factor in elderly AML patients and this is a notable result which can lead to future investigation.

In conclusion, we report that the prognostic risk classification based on CAR can easily stratify elderly patients with newly diagnosed AML.

## Supplementary Information

Supplementary Information.

## Data Availability

The datasets used and/or analyzed during the current study available from the corresponding author on reasonable request.
