# Supplementary material for: High CRP-albumin ratio predicts poor prognosis in transplant ineligible elderly patients with newly diagnosed acute myeloid leukemia
Source: Sci Rep. 2022 May 25;12:8885. doi: 10.1038/s41598-022-12813-1 (PMC9133033; doi:10.1038/s41598-022-12813-1)
Supplement: Supplementary file 1 — Supplementary Information. [file 41598_2022_12813_MOESM1_ESM.pdf]

Supplementary information

High CRP-Albumin Ratio Predicts Poor Prognosis in Transplant Ineligible Elderly Patients with Newly Diagnosed Acute Myeloid Leukemia

Hajime Senjo[1], Masahiro Onozawa[1], Daisuke Hidaka[2], Shota Yokoyama[1], Satoshi Yamamoto[3], Yutaka Tsutsumi[4], Yoshihito Haseyama[5], Takahiro Nagashima[6], Akio Mori[7], Shuichi Ota[2], Hajime Sakai[8], Toshimichi Ishihara[9], Takuto Miyagishima[10], Yasutaka Kakinoki[11], Mitsutoshi Kurosawa[12], Hajime Kobayashi[13], Hiroshi Iwasaki[14], Daigo Hashimoto[1], Takeshi Kondo[6] and Takanori Teshima[1].

[1] Department of Hematology, Faculty of Medicine, Hokkaido University, Sapporo, Japan
[2] Department of Hematology, Sapporo Hokuyu Hospital, Sapporo, Japan
[3] Department of Hematology, Sapporo City General Hospital, Sapporo, Japan
[4] Department of Hematology, Hakodate Municipal Hospital, Hakodate, Japan
[5] Department of Hematology, Tonan Hospital, Sapporo, Japan
[6] Department of Hematology, Japanese Red Cross Kitami Hospital, Kitami, Japan
[7] Blood Disorders Center, Aiiku Hospital, Sapporo, Japan
[8] Department of Hematology, Teine Keijinkai Hospital, Sapporo, Japan
[9] Department of Hematology, Kin-ikyo Chuo Hospital, Sapporo, Japan
[10] Department of Hematology, Kushiro Rosai Hospital, Kushiro, Japan
[11] Department of Hematology, Asahikawa City Hospital, Asahikawa, Japan
[12] Department of Hematology, Hokkaido Cancer Center, Sapporo, Japan
[13] Department of Hematology, Obihiro Kosei General Hospital, Obihiro, Japan
[14] Department of Hematology, Sapporo Kosei General Hospital, Sapporo, Japan

Correspondence Author

Hajime Senjo
Department of Hematology, Faculty of Medicine, Hokkaido University, Sapporo, Japan
N15 W7, Kita-ku, Sapporo, 060-8638, Japan
Telephone: 81-11-865-0111, FAX: 81-11-865-0201 E-mail: hajimesenjo@gmail.com

Figure legends

Figure S1. Age distribution of AML patients registered in Hokkaido Leukemia Network from 2010 to 2019 (n=517)

Figure S2. Study consort diagram of the current study

Figure S3. Receiver Operatorating Characteristic (ROC) curves according to CAR (A), CRP (B) and 1/Alb (C) with regarding death as positive finding and Kaplan-Meier plots of OS stratified these cut off values according to CAR (D), CRP (E), and Alb (F).

Table S1. Risk classification of acute myeloid leukemia based on validated cytogenetic and molecular abnormalities according to National Comprehensive Cancer Network (NCCN) Guidelines Version 3. 2017

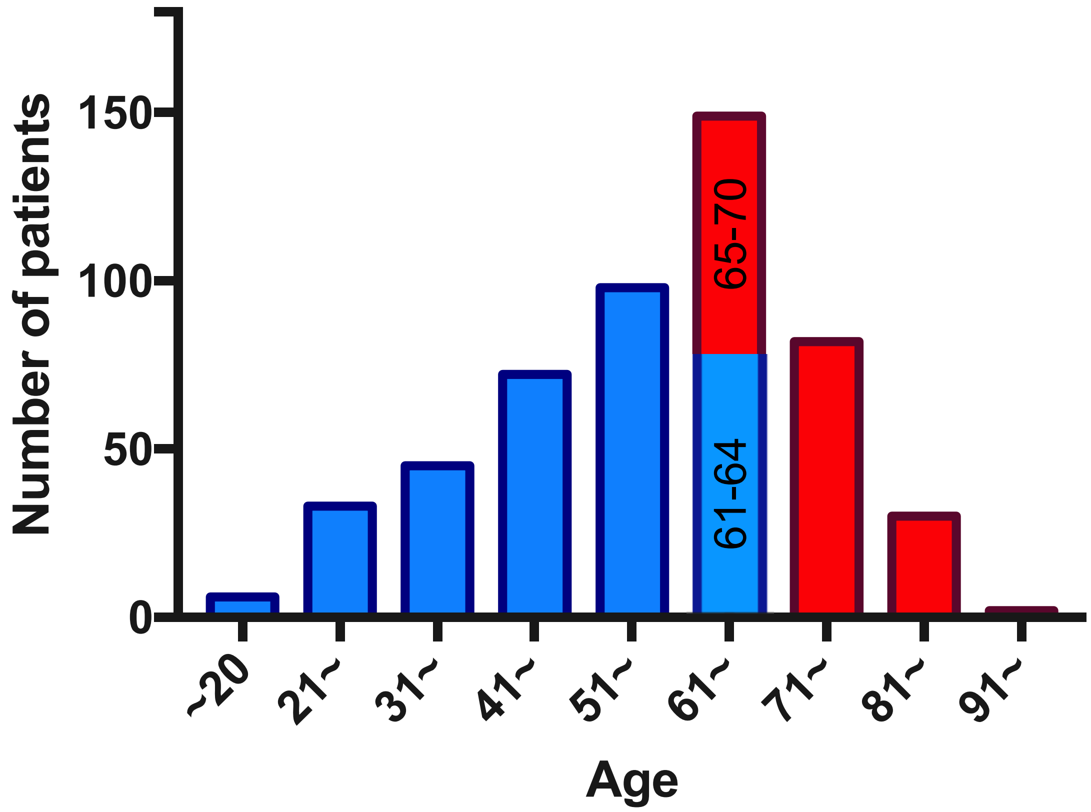

Fig S1. Age distribution of AML patients registered in Hokkaido Leukemia Network from 2010 to 2019 (n=517). Red bar represents patients with age ≧ 65 (n=196, 37.9%).

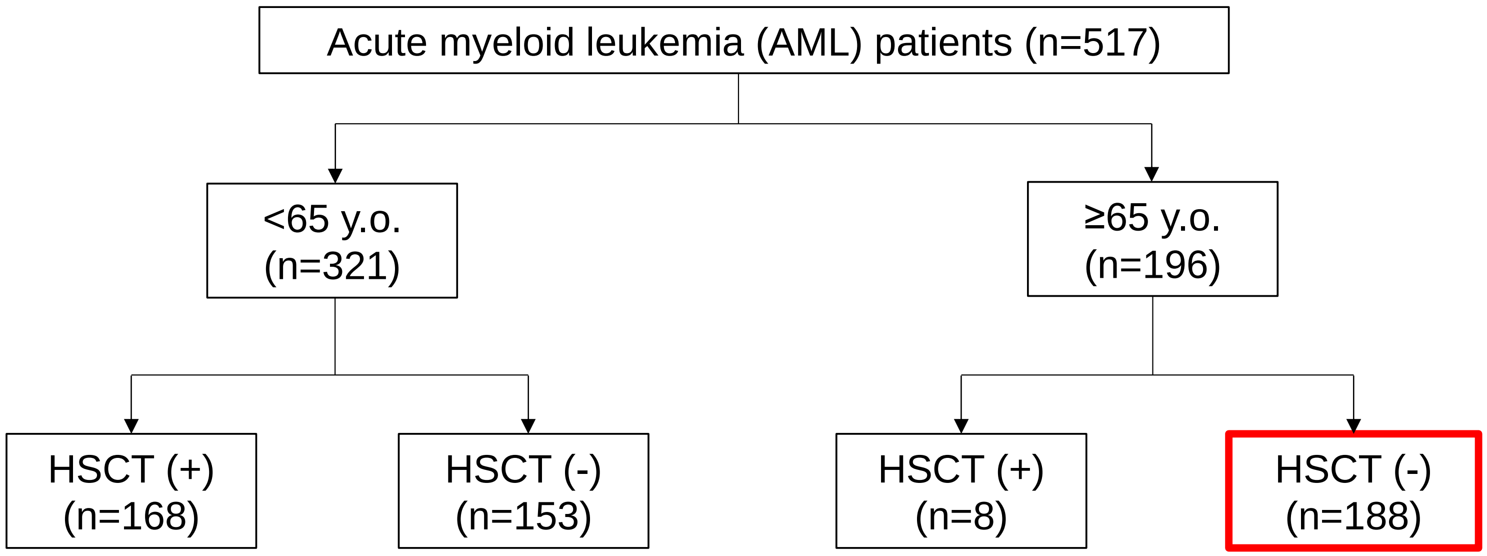

Fig S2. Study consort diagram of the current study

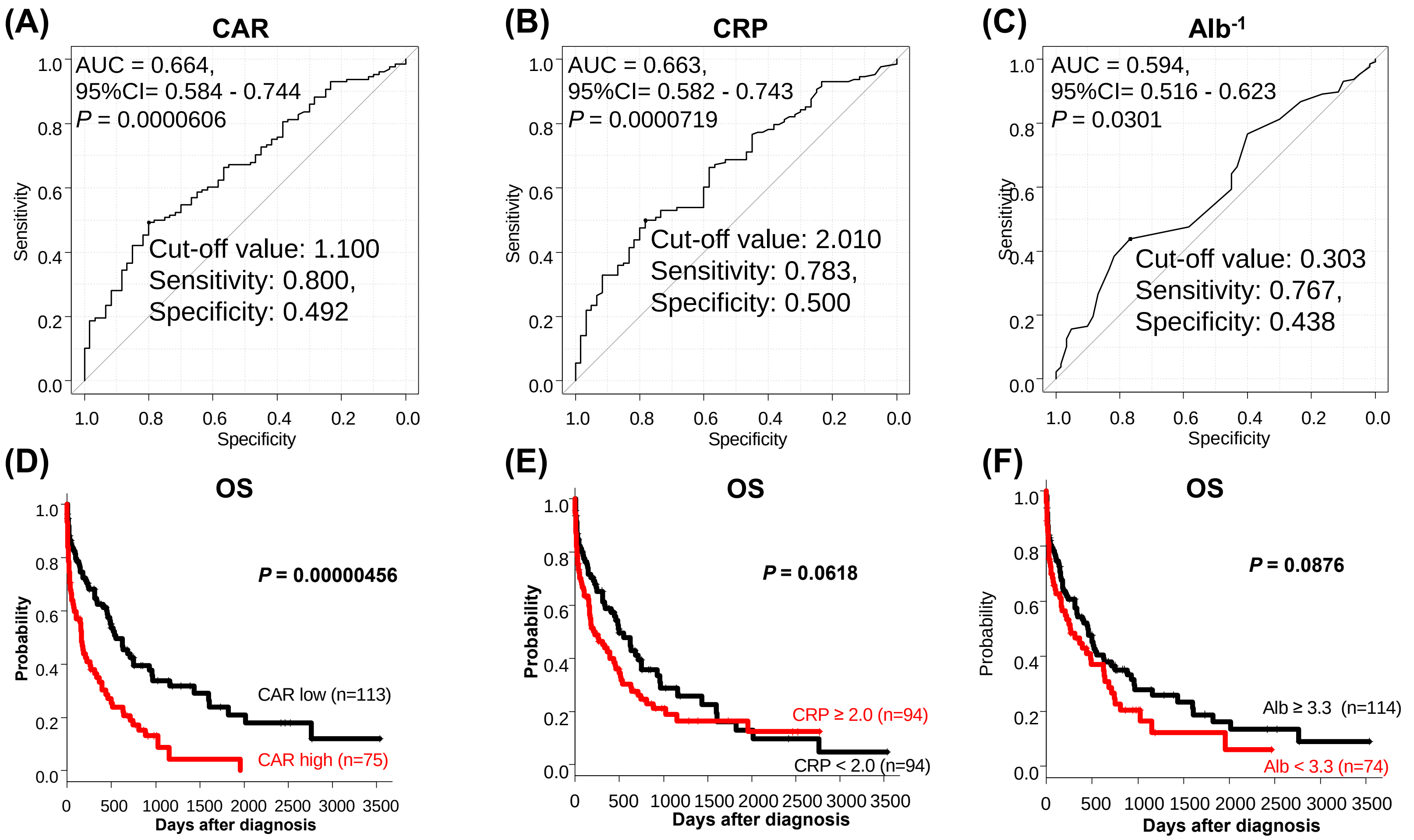

Fig S3. Receiver Operatorating Characteristic (ROC) curves according to CAR (A), CRP (B) and 1/Alb (C) with regarding death as positive finding and Kaplan-Meier plots of OS stratified these cut off values according to CAR (D), CRP (E), and Alb (F).

Risk status	Cytogenetics	Molecular abnormalities
Favorable	Core binding factor: inv(16) or t(16;16) or t(8;21) or t(15;17)	Normal cytogenetics:<br>NPM1 mutation in the absence of FLT3-ITD or isolated biallelic (double) CEBPA mutation
Intermediate	Normal cytogenetics<br>+8 alone<br>t(9;11)<br>Other non-defined	Core binding factor with KIT mutation
Poor	Complex (≥3 clonal chromosomal abnormalities)<br>Monosomal karyotype<br>-5, 5q-, -7, 7q-<br>11q23 - non t(9;11)<br>inv(3), t(3;3)<br>t(6;9)<br>t(9;22)	Normal cytogenetics:<br>with FLT3-ITD mutation<br>TP53 mutation

Table S1. Risk classification of acute myeloid leukemia based on validated cytogenetic and molecular abnormalities according to National Comprehensive Cancer Network (NCCN) Guidelines Version 3. 2017
